# Multiclass Classifier for P-Glycoprotein Substrates, Inhibitors, and Non-Active Compounds

**DOI:** 10.3390/molecules24102006

**Published:** 2019-05-25

**Authors:** Liadys Mora Lagares, Nikola Minovski, Marjana Novič

**Affiliations:** 1 Theory Department, Laboratory for Cheminformatics, National Institute of Chemistry, 1000 Ljubljana, Slovenia; liadys.moralagares@ki.si (L.M.L.); nikola.minovski@ki.si (N.M.); 2 Jožef Stefan International Postgraduate School, 1000 Ljubljana, Slovenia

**Keywords:** P-glycoprotein, counter-propagation artificial neural networks (CP ANN), multiclass classifier, substrates, inhibitors

## Abstract

P-glycoprotein (P-gp) is a transmembrane protein that actively transports a wide variety of chemically diverse compounds out of the cell. It is highly associated with the ADMET (absorption, distribution, metabolism, excretion and toxicity) properties of drugs/drug candidates and contributes to decreasing toxicity by eliminating compounds from cells, thereby preventing intracellular accumulation. Therefore, in the drug discovery and toxicological assessment process it is advisable to pay attention to whether a compound under development could be transported by P-gp or not. In this study, an in silico multiclass classification model capable of predicting the probability of a compound to interact with P-gp was developed using a counter-propagation artificial neural network (CP ANN) based on a set of 2D molecular descriptors, as well as an extensive dataset of 2512 compounds (1178 P-gp inhibitors, 477 P-gp substrates and 857 P-gp non-active compounds). The model provided a good classification performance, producing non error rate (NER) values of 0.93 for the training set and 0.85 for the test set, while the average precision (AvPr) was 0.93 for the training set and 0.87 for the test set. An external validation set of 385 compounds was used to challenge the model’s performance. On the external validation set the NER and AvPr values were 0.70 for both indices. We believe that this in silico classifier could be effectively used as a reliable virtual screening tool for identifying potential P-gp ligands.

## 1. Introduction

The P-glycoprotein (P-gp or ABCB1) is a transmembrane protein belonging to the ATP-binding cassette family of transporters (ABC-transporters). P-gp is an efflux pump that actively transports a large number of compounds (structurally diverse) out of the cell using the energy provided by the hydrolysis of ATP [1,2]. Efflux pumps are considered as a first line of defense against toxicants; they decrease the toxicity due to xenobiotic exposure by restricting their absorption and transport, thus preventing their intracellular accumulation.

P-gp has a great influence on the ADMET (absorption, distribution, metabolism, excretion and toxicity) properties of drugs and toxins. This is evidenced by its widespread expression in the small intestine, liver, colon, kidneys, placenta and the blood-brain-barrier (BBB) [3], i.e., tissues that perform an excretory or barrier function. P-gp is also involved in the multidrug resistance (MDR) phenomenon [4,5], whereby drugs are pumped out of the cell and their concentration is lowered at the intracellular target site. This was assessed to be a pivotal reason for chemotherapeutic failures in the treatment of various cancers, as P-gp is commonly over-expressed in tumour cell lines [6].

Taking into account the role of P-gp in drug absorption and disposition, special attention should be paid on both the substrate and inhibitory properties of new compounds. Inhibitors are particularly interesting for drug-drug interactions; there have been several cases reported where the co-administration of a P-gp inhibitor and a P-gp substrate frequently led to an increased blood concentration of the substrate, which in turn can cause serious side effects [7,8]. Considering the major role of ABC transporters in reducing the toxic events related to drug-drug interactions, the elucidation of ligand–transporter interactions for the effective prediction of a ligand’s toxicity and safety needs much more attention and requires more complex strategies.

Nowadays, the food and drug administration (FDA) recommends a standardized set of experiments to assess the likelihood of a compound to interact with P-gp and the breast cancer resistance protein (BCRP/ABCG2) [9], evidencing that in silico models for the prediction of a compound to interact with P-gp are of high importance in the early stages of drug discovery, development, and toxicological assessment processes.

Many studies have been performed with the aim of identifying P-gp substrates or to develop more potent, selective and specific P-gp inhibitors; however, the huge variety of ligands (polyspecificity or promiscuity of P-gp) makes difficult the effective design of new P-gp interacting compounds [10]. Put differently, most of the P-gp inhibitors interact with the same binding site as the P-gp substrates, even when substrates and inhibitors have different biological purposes, indicating that both classes could share multiple structural similarities, which makes the differentiation of compounds belonging to one class from another extremely difficult.

The first P-gp studies started with ligand-based approaches (e.g., Beck et al. [11], Seeling et al. [12], Ichiro et al. [13], and Dearden et al. [14]), which extended to the structure-based level when the first X-ray structure of a mouse P-gp became available (PDB ID: 3G5U) [15]. Several methods available for ligand-based design have been applied, including ligand-based pharmacophore modelling [16], linear and non-linear classification algorithms [17], as well as supervised and unsupervised artificial neural networks [18]. These methods were mainly applied for constructing in silico models relating the P-gp inhibitory activity of the compounds and a set of very basic physicochemical parameters (e.g., lipophilicity, H-bonding, aromatic rings and charge).

During the last 10 years, a variety of P-gp classification models were developed focusing on large datasets with the purpose of screening compound libraries. Some of the most relevant contributions are related to the work of Broccatelli et al. [19] and Chen et al. [20]. In both studies, large datasets of thousands of compounds were used to develop models for classification and prediction of P-gp inhibitors; according to their results, the compound’s lipophilicity expressed as the logP was found to be a crucial parameter for distinguishing between P-gp inhibitors and non-inhibitors.

Besides P-gp inhibitor/non-inhibitor classification models, different pharmacophore models have been developed as well. It is widely known that pharmacophore models can significantly contribute towards understanding probable P-gp-ligand interactions by assessing the relevant pharmacophoric features. However, taking into account the high structural diversity of the known P-gp ligands, the pharmacophore modelling concept did not lead to a better understanding of their polyspecificity. Nevertheless, some of the models gave good results in identifying new ligands as in the study of Palmeira et al. [21] in which starting from a DrugBank screening, 12 compounds were identified to increase the intracellular accumulation of Rhodamine-123, an in vitro biologically confirmed P-gp substrate.

In the case of classifying P-gp substrates and non-substrates, the studies of Wang et al. [22], and Li et al. [23] can be considered as dealing with the largest datasets reported so far (comprised of 332 and 723 compounds, respectively). Their results link substrate activity to a set of very common physicochemical properties, such as molecular weight and water solubility.

For structure-based P-gp modelling, several molecular docking studies of ligands based on the available murine crystallographic data (PDB ID: 3G5U) [15] have been performed with the goal of understanding the molecular interactions that determine ligands binding to P-gp, as demonstrated in the studies of Dolghih et al. [24] and Ferreira et al. [25].

According to the FDA recommendations, “all investigational drugs should be evaluated in vitro to determine whether they are a potential substrate of P-glycoprotein” [9]. P-gp inhibitors and substrates are measured with different assay protocols. If a bidirectional transport assay is used, based on a polarized epithelial monolayer that overexpresses P-gp, one can distinguish the P-gp substrates from inhibitors by the net flux above or below 2, respectively [26]. However, the majority of data available in the literature often lacks this information. Consequently, one has to be aware of promiscuous nature of P-gp and different assay protocols when merging data from different sources.

Here we show the development and performance of a multiclass classifier able to differentiate substrates and inhibitors and not only between active/non-active compounds. The development of the classifier was based on the experimental data available (interaction with P-gp) for the compounds forming the dataset. It is clear that compared to the experimental drug design methods, in silico methods are faster, cheaper, more efficient and high-throughput in screening, with reduced labour and use of animals [27]. Therefore, the aim of the present study was to develop an efficient in silico screening tool capable of providing a rapid and cost-effective platform for the in silico identification of potential P-gp substrates, inhibitors or non-active compounds, which could effortlessly be used in drug discovery and toxicological assessment processes. The identification of potential P-gp substrates and inhibitors is of great concern, not only for the development of agents able to counteract the mechanisms of multidrug resistance (MDR reversal agents), but also for eliminating drug candidates that are P-gp substrates.

## 2. Results and Discussion

**Figure 1:**
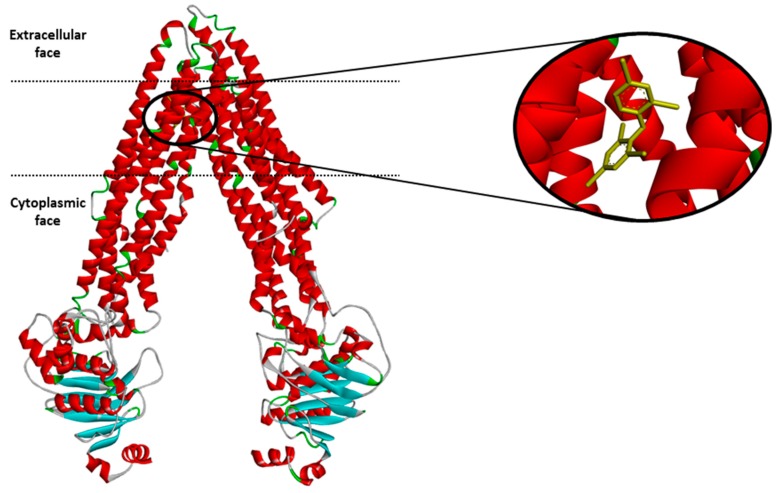
Structure of the mouse P-glycoprotein based on the 4XWK PDB coordinates with the ligand BDE-100.

The dataset encompasses the compounds that were tested on the interactions with P-gp (2512 compounds, see Supplementary material, Excel file S1). They were grouped into three classes: P-gp inhibitors, substrates and non-active molecules. An example of the P-gp structure with a ligand is shown in Figure 1 below.

### 2.1. Descriptors

The genetic algorithm-based [28] optimization procedure resulted in the selection of 26 molecular descriptors (see Table 1), which were used in the construction of the classification model. Among these 26 descriptors, there are six 2D atom pairs; three CATS 2D descriptors, three Functional group counts and three 2D autocorrelations; two ring descriptors and two P_VSA-like descriptors; one atom-type E-state index, one atom-centred fragment, one 2D matrix-based descriptor, one information index, one connectivity index, one walk and path count and one constitutional index.

Most of the selected descriptors (more than half of them) are count descriptors, providing information about occurrences, or specifying the presence/absence of predefined structural features in the molecule, such as functional groups, augmented atoms, pharmacophore point pairs, atom pairs, presence of rings, and walk and path counts. Some of these count descriptors help in discriminating cyclic compounds from acyclic ones and reflect the local geometrical environment in complex cyclic systems. Therefore, they contribute to a deeper understanding of the structural complexity of the molecules.

Other molecular descriptors more present in the selected set are the autocorrelation descriptors. They describe how the property considered is distributed along the topological structure of the molecule, e.g., the ATS descriptor corresponds to a decomposition of the square molecular property in different atomic contributions. On the other hand, the P_VSA like descriptors included provide information about the amount of Van der Waals surface area (VSA) having a particular property P in a certain range. The properties taken into account in this case are the log P and ppp (potential pharmacophore points) hydrogen-bond donor. The selection of these two properties is in agreement with previous in silico studies which have suggested essential chemical properties like lipophilicity [29,30] and hydrogen bond acceptor/donor [31] as playing important roles in the interaction of ligands with P-gp.

The selected set of descriptors also includes one simple constitutional descriptor, i.e., the percentage of H atoms, as well as some relevant indices, such as the average connectivity index and the information index, which give information about the shape of the molecule, and the atom-type E-state index that gives topological and electronic information related to particular atom types in the molecule.

### 2.2. Classification Model

Sensitivity, specificity and precision are commonly used to evaluate classifiers, but in the case of multiclass problems they do not give a global evaluation of the classification quality; they just give information about the classifier performances on each specific class. Therefore, global indices derived from primary class measures [32], namely sensitivity and precision, have been proposed. The average sensitivity, also known as non error rate (NER), and average precision (AvPr) are calculated as the arithmetic mean of sensitivity and precision values of the G classes. An analogoue global measure based on specificity values has never been proposed, probably due to the specificity bias in relation to the number of classes G.

(1) $$NER=\frac{\sum_{g\;=1}^{G}Sn_{g}}{G}$$

(2) $$AvPr=\frac{\sum_{g=1}^{G}Pr_{g}}{G}$$

Regarding accuracy, one of the most common used classification indices in the literature, it is known to be influenced by the presence of unbalanced classes, as in this case; it is biased towards the most numerous class and for this reason it is not consider in this study to evaluate the classifier’s performance.

Different models were built modifying the network size from 20 × 20 to 45 × 45 neurons and the number of learning epochs from 100 to 2000. The minimum and maximum learning rates were set to 0.001 and 0.6, respectively. The best classification performance was obtained for models with a network dimension of 43 × 43 neurons.

**Figure 2:**
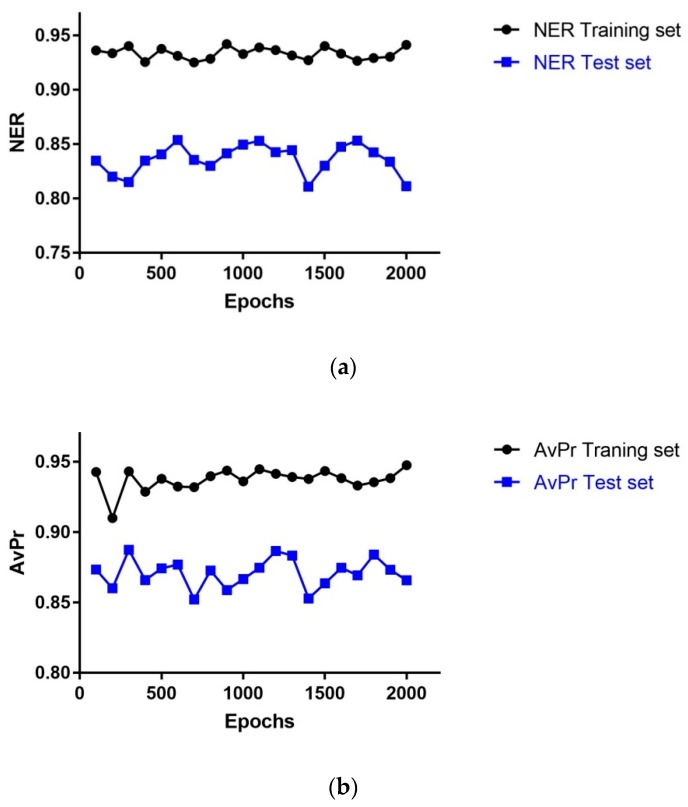
Statistical performance of models with dimension 43 × 43 neurons depending on the number of learning epochs: (**a**) NER versus number of Epochs; (**b**) AvPr versus number of Epochs.

The statistical performance of the models with dimension 43 × 43 neurons depending on the number of learning epochs is shown in Figure 2. The highest NER value for the Test set (TE)(NER_TE_ = 0.85) was obtained using 600 epochs, whereas the highest AvPr value for the TE set (AvPr_TE_ = 0.88) obtained using 300 epochs; the second highest AvPr value (0.87) was got with 600 epochs; hence, the optimal number of learning epochs for the model was set to 600 as with 300 epochs the NER has the minimum value in the curve for the TE set.

After the optimization of the models, the model with the best classification ability was selected. The statistical performance of this model is presented in Table 2, the confusion matrix in Table 3 and the network parameters in Table 4. The global performance of the model based on the training set (TR) showed a NER value of 93.10% and AvPr of 93.22%. For the TE set the NER was 85.37% and AvPr 87.68%. For the external validation set the NER and AvPr values were of 70.21% and 70.08 %, respectively, demonstrating a good classification performance.

Looking at the performance of the classifier in each specific class, the results for the sensitivity were quite good with values greater than 91% in the TR set and above 80% in the TE set. The specificity was also good with values greater than 90% in both TR and TE sets. In addition, the MCC values showed the capability of the model for classifying both positive and negative objects, with values greater than 0.77 in the TR and TE sets. The model was challenged using the V set, which provided sensitivity values greater than 65% and specificity values greater than 83%. The MCC values were around 0.50 evidencing a good predictive performance of the model.

**Figure 3:**
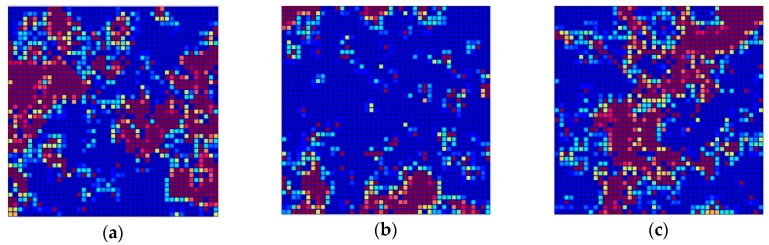
Distribution of objects in the Kohonen top-map. Neurons coloured in red represent the position of the objects belonging to the corresponding class: (**a**) Inhibitors (I), (**b**) Substrates (S), and (**c**) Non-active compounds (NA).

Figure 3 shows the distribution of the 1,786 structurally diverse compounds of the TR set in the Kohonen top-map. The separation of P-gp substrates, inhibitors and non-active compounds is rather good. P-gp inhibitors are mainly clustered in the right and left side of the network, while P-gp substrates are clustered in the upper and inner part of the top-map. P-gp non-active compounds are located in the central part of the network drawing a diagonal from the inner left part to the upper right part of the top-map; however, some of the compounds from each class are located outside the clusters formed. A complete separation of P-gp substrates, inhibitors, and non-active compounds was not possible, even with larger dimensions of the network due to the high structural similarities between compounds belonging to different classes.

### 2.3. Applicability Domain of the Model

The AD of the model in this study was analysed using the ED between the molecules and the central neuron of the neural network. This metric gives the possibility of comparing the TR and TE sets chemical coverage with respect to false predicted chemical space.

In the model developed, the target value of 1 was set for compounds belonging to one class and zero for compounds not belonging to that class. The predicted response values are expressed as continuous values in the interval between 0 and 1, so the threshold for the separation of the objects belonging, or not, to the specific class was established equal to 0.5. If a compound has a predicted value > 0.5, then the compound is classified as belonging to the class under study; if it is < 0.5 then it is classified as not belonging to the class under study. The data closer to the threshold can be determined by our model as correctly predicted but they are less reliable. The area closer to the threshold is the uncertainty area because here the results of the model contain very high uncertainty in prediction.

**Figure 4:**
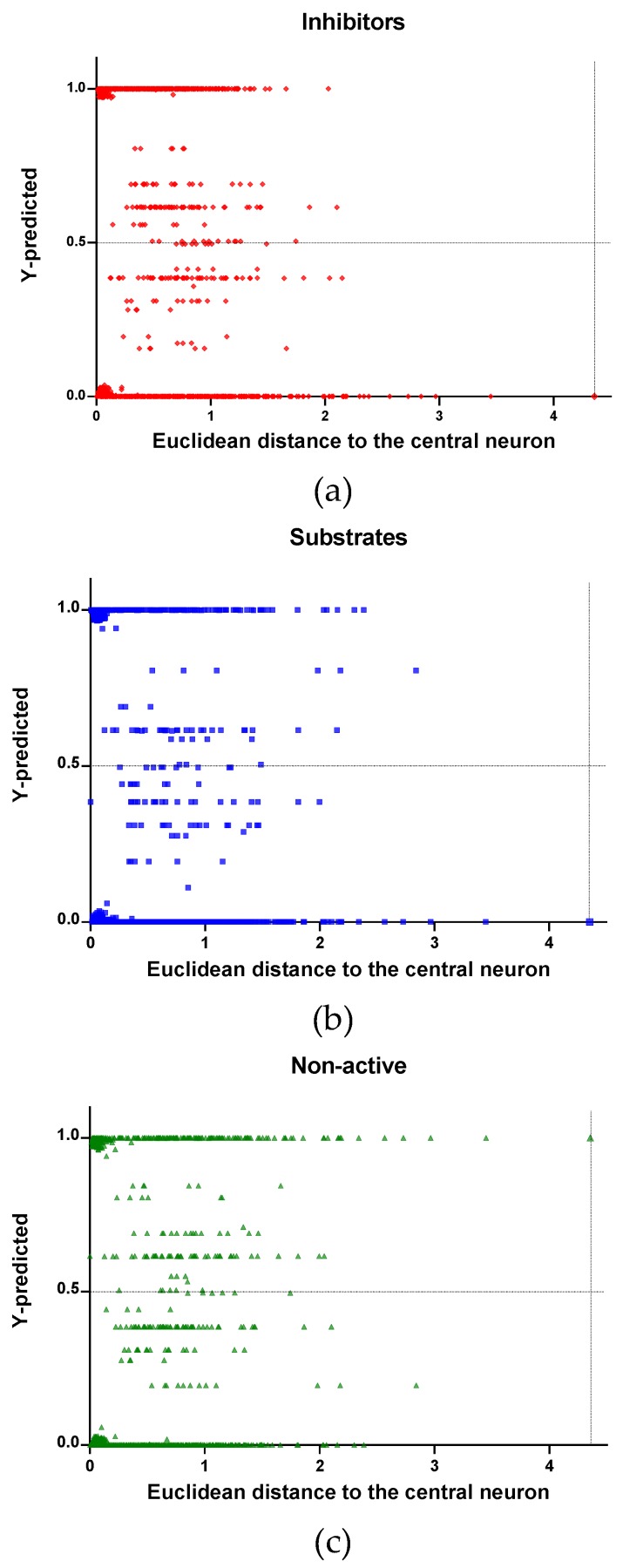
Qualitative assessment of the applicability domain for the selected mode: Training set (TR): (**a**) Inhibitors, (**b**) Substrates and (**c**) Non-active.

**Figure 5:**
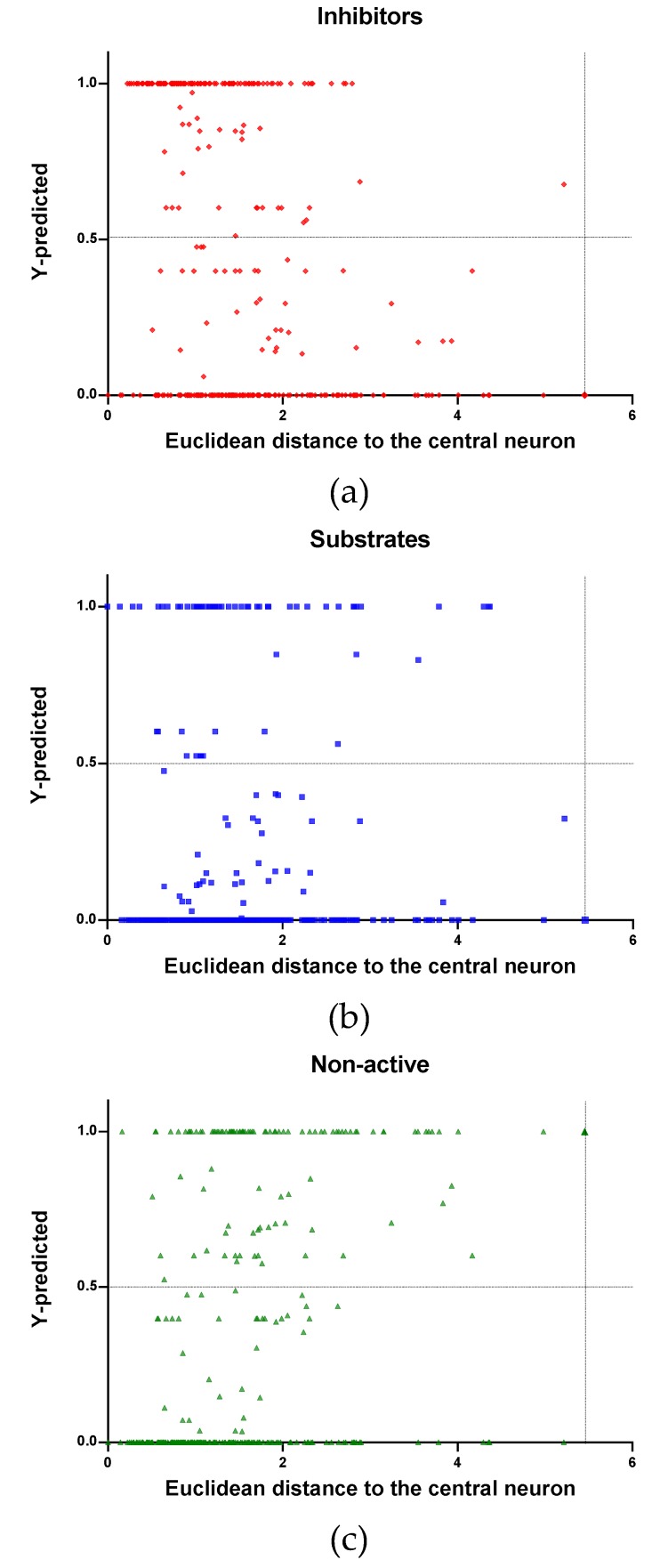
Qualitative assessment of the applicability domain for the selected mode: Test set (TE): (**a**) Inhibitors, (**b**) Substrates and (**c**) Non-active.

**Figure 6:**
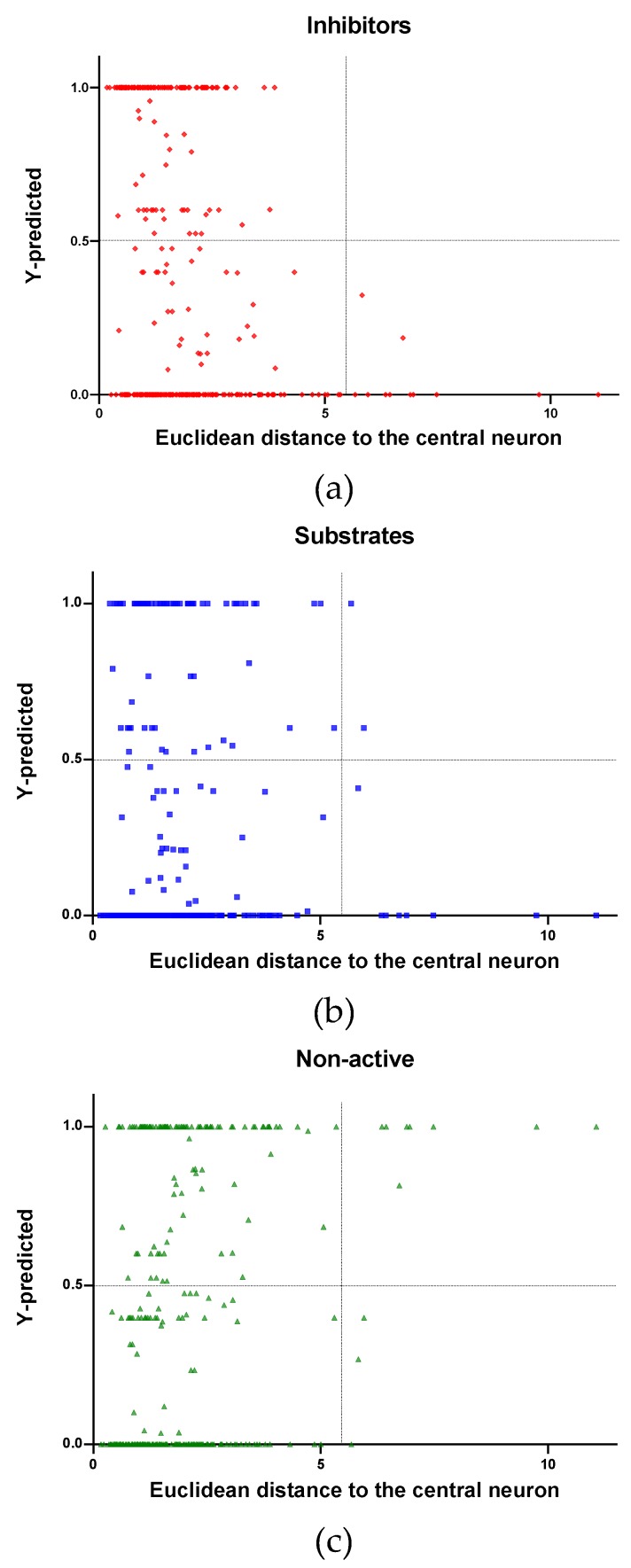
Qualitative assessment of the applicability domain for the selected mode: Validation set (V): (**a**) Inhibitors, (**b**) Substrates and (**c**) Non-active.

In Figure 4, Figure 5 and Figure 6 is plotted the ED to the central neuron versus the predicted values in each class, for the TR, TE and V sets, respectively. The ED here shows the similarity or dissimilarity between the compounds. The maximal value of the ED characterizes the boundaries of the model under study. In these plotsthe area close to the threshold (0.5) represents the uncertainty zone of the prediction. The chemicals predicted as false or with high level of uncertainty are grouped here. Thus, the predicted P-gp inhibitors (Figure 4a, Figure 5a and Figure 6a), substrates (Figure 4b, Figure 5b and Figure 6b), or non-active compounds (Figure 4c, Figure 5c and Figure 6c) that are closer to the edge of class (Y = 1) and (Y = 0) are assumed to have better prediction accuracy comparing to those located in the middle, near the threshold value 0.5.

In Figure 4 and Figure 5 each plot has a vertical dotted line indicating the maximum values of ED resulted in the TR and TE sets, respectively. In the TR set the EDs were mostly kept within low values. Hence, the largest ED distance to the the central neuron (5.45) was obtained with the TE set and it is the distance considered for the analyss in this discussion. In Figure 6, corresponding to the V set results, the dotted line was set at the distance obtained with the TE set because is the largest one obtained during the construction of the model and it can be used as a reference point to better analyse the boundaries of the model.

**Figure 7:**
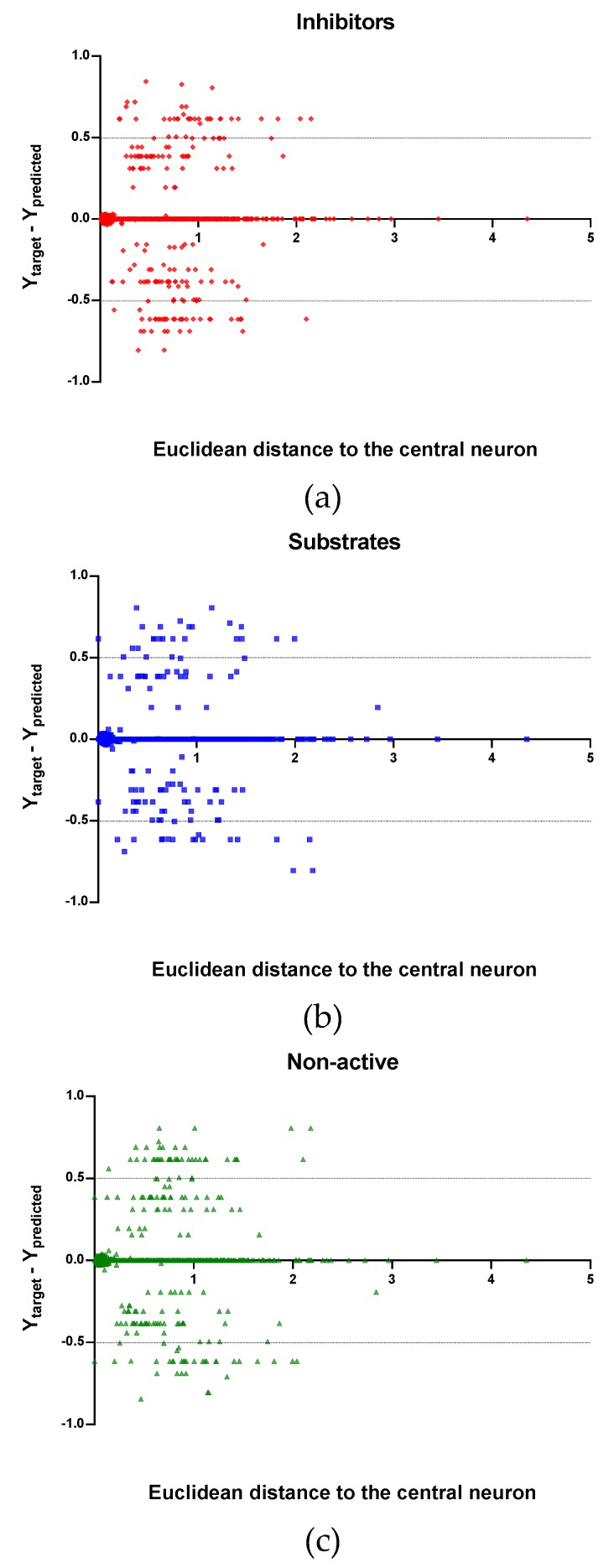
Plot of the EDs to the central neuron versus (Ytarget–Ypredicted) for the Training set (TR): (**a**) Inhibitors, (**b**) Substrates and (**c**) Non-active.

**Figure 8:**
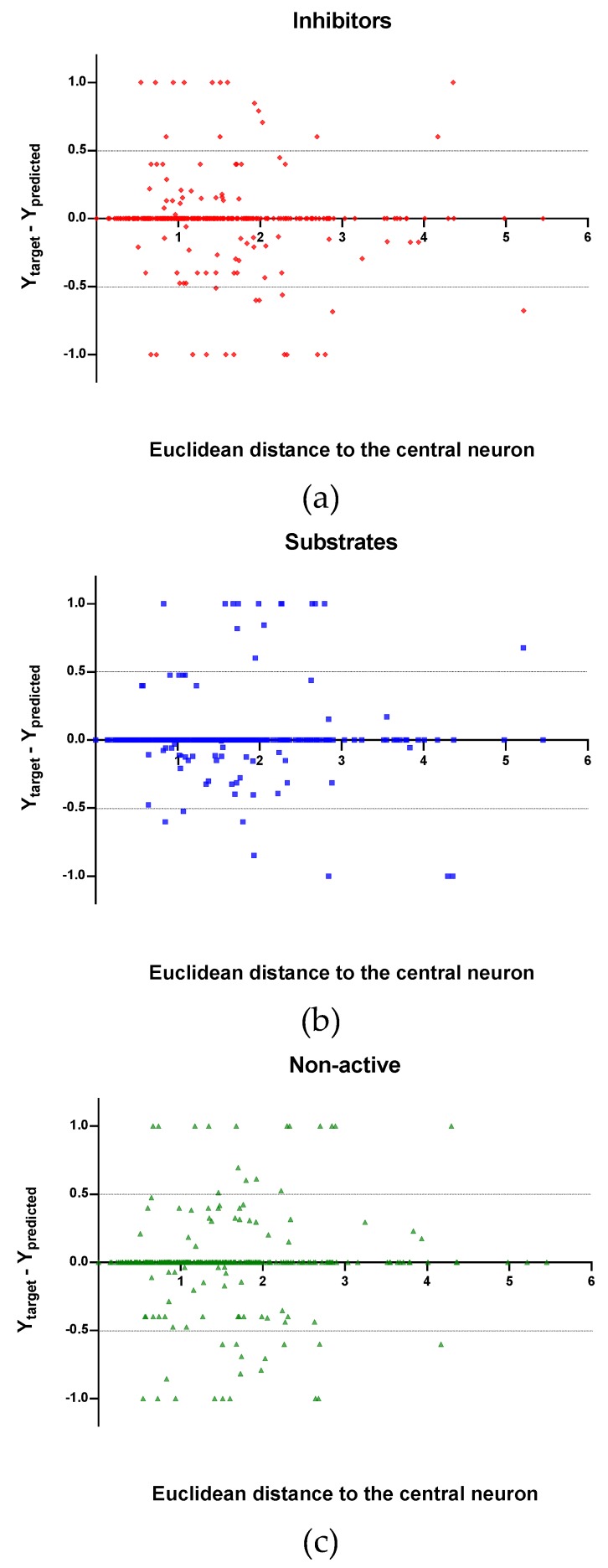
Plot of the EDs to the central neuron versus (Ytarget—Ypredicted) for the Test set (TE): (**a**) Inhibitors, (**b**) Substrates and (**c**) Non-active.

**Figure 9:**
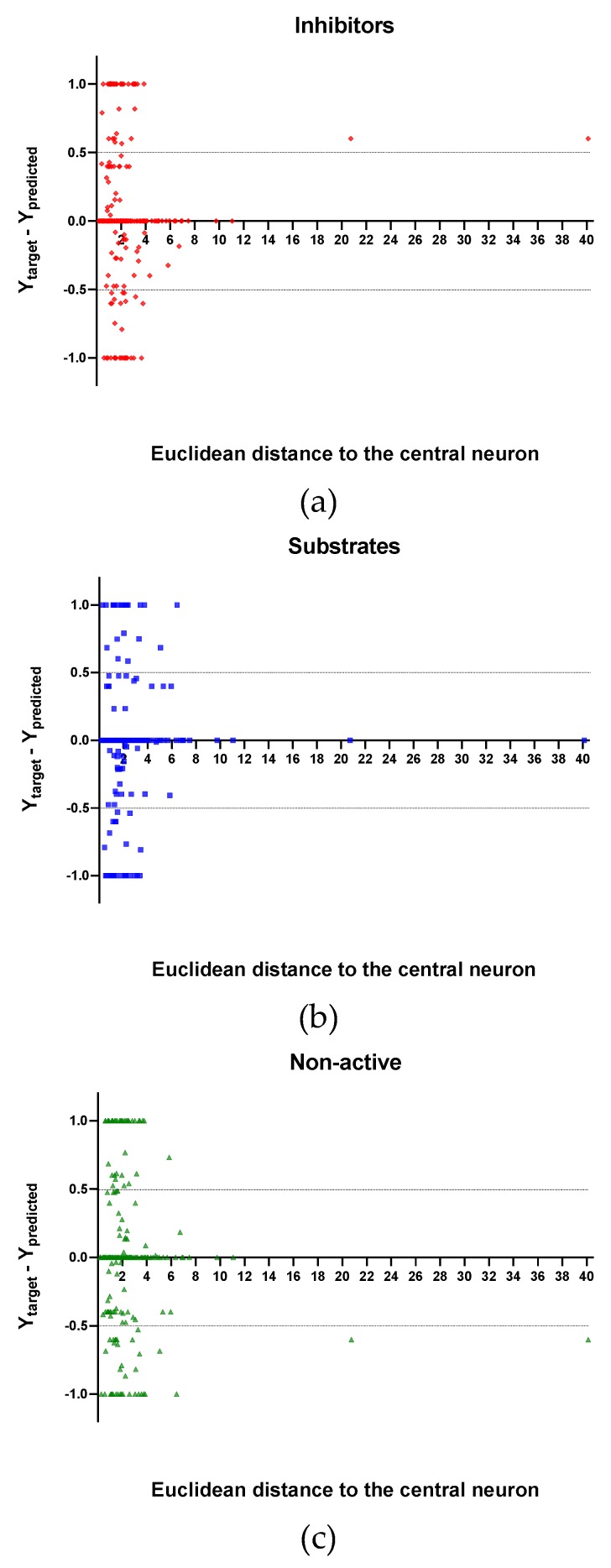
Plot of the EDs to the central neuron versus (Ytarget – Ypredicted) for the Validation set (V): (**a**) Inhibitors, (**b**) Substrates and (**c**) Non-active.

Observing the descriptor values of the compounds with the largest ED to the central neuron in the TE, it can be noticed that one count descriptor, one atom-centred fragment descriptor (F04[C-P], H-048) and two autocorrelation descriptors (MATS6v, GATS4s) have the highest values in the data set. The two compounds with the largest ED in the TE set are Phosmet and Triphenylphosphane, both compounds share the presence of a Phosphorous atom in their structure (see Supplementary material, Figure S1), which can explain the high values for the Frequency of C –P at topological distance 4 descriptor. Nonetheless, the large values of the ED in the model is not evidence of incorrect prediction. In Figure 7, Figure 8 and Figure 9, the plots show the distribution of the true predicted and false predicted compounds relative to the threshold (0.5). All values above and below the threshold are the false predicted by the model. In the plots it can be noted that some of the false predicted compounds are within the shortest ED to the central neuron.

In the V set, thirteen compounds have ED larger than the ED value taken as reference point. These thirteen compounds have in common the presence of high electronegative atoms in their structure like chlorine, fluorine, bromine and sulfur (see Supplementary material, Figure S2). However, just four out of thirteen were falsely predicted by the model. This could be due to the uncertainties present in the predictions. Some predictions may fall within the defined AD of the model but be unreliable due to properties and features not accounted for by the model. On the other hand, a chemical falling outside the defined AD may still exhibit the response being modelled, because it brings out this response by a mechanism not accounted by the model under study.

In these plots the majority of correctly predicted compounds are concentrated within the shorter ED, suggesting this as the region where predictions are expected to be reliable.

## 3. Materials and Methods

### 3.1. Dataset

The compounds present in the dataset were collected mainly from the admetSAR database [33]. This database is a compilation of diverse chemicals gathered from different literature sources associated with known ADMET profile. The data was extracted from the original studies [19,20,22] as SMILES notations together with their corresponding experimentally determined P-gp class (inhibitors, non-inhibitors, substrates, and non-substrates). An additional set of P-gp substrates was retrieved from the work of Li et al. [23].

In order to enlarge the data set and to extend the chemical space, we collected and included in our data set compounds that derive from different references which use different kinds of experimental assay for assessing the P-gp class. Hence, data pre-processing was required to detect duplicate compounds and compounds with both experimental classes (or overlapping classified compounds) before the construction of the model. The P-gp non-inhibitor and non-substrate compounds were merged into the non-active class. The overlap of negative compounds in both sets was desirable (see Supplementary material, Figure S3), so they were included in the non-active class, while all other overlaps might introduce uncertainty into the model (S/I = 42 S/NI = 29 I/NS = 10; S: substrate I: inhibitor NI: non-inhibitor NS: non-substrate), so they were removed. The final dataset constitutes 2512 structurally diverse compounds, e.g., acridone derivatives, flavonoids, azoles, antidepressants of the selective serotonin reuptake inhibitor (SSRI) class, persistent organic pollutants (POPs), β-lactam antibiotics, and benzodiazepines, among others, which can be separated into three main classes, i.e., 1178 P-gp inhibitors, 477 substrates, and 857 non-active compounds.

In this study, 42 compounds were found to belong to the substrate and inhibitor class. These compounds were removed from the data set because for the purposes of our study we wanted to include just well-defined compounds in each class. Some drugs can belong to multiple P-gp classes [26], however, the available experimental assays use different criteria to classify Pgp-interacting compounds, which leads to diverse reports of their class. Differences in reports of the Pgp class is quite common and it could be due to the variety of available assays and also to the criteria used for determining Pgp activity (threshold used) in each individual assay. The promiscuity of the Pgp transporter itself and its interacting ligands is other possible reason.

The data curation was mainly done utilizing the Pipeline Pilot software 9.2 [34]. Since the data set was collected from different literature sources the SMILES notations present had a high level of heterogeneity. In order to facilitate the data curation, it was necessary to convert the original SMILES notations into a uniform representation, running a Pipeline Pilot protocol. The protocol used includes the canonical smiles component, which adds canonical smiles as a new property to the dataset. All newly generated SMILES, along with their Pgp class label, were then combined within a single. sdf file format. Duplicate compounds were identified and removed from further analysis running a pipeline pilot protocol which includes the remove duplicate molecules component. In this component the canonical smiles was set as a filter to find duplicates. In addition, the compounds that were classified as belonging to more than one class, defined as overlapping compounds, were also discarded from the analysis. After running the pipeline pilot protocol there were still some duplicate compounds present in the dataset. Removing the remaining duplicates and overlapping compounds was done manually based on the descriptor values for the molecules.

Prior to the modelling, the entire dataset was divided into training (TR), test (TE) and validation (V) sets, which comprise 1786, 341, and 385 compounds, respectively, utilizing the Kohonen mapping as implemented in the CPANNatNIC software [35].

### 3.2. Descriptors Calculation

For the entire dataset, 2D molecular descriptors were calculated by the software Dragon 7.0 [36]. Initially, a total of 1229 molecular descriptors were calculated, and their values were normalized according to the Equation (3):

(3) $$x_{i}^{norm}\;=\;\frac{x_{i}\;-\;\bar{x}}{s_{x}}$$

where $x_{i}^{norm}$ represents the normalized value of $x_{i}$ descriptor for the *i*^th^ molecule, the $\bar{x}$ is the average of all descriptor *x_i_* values in the data set, while $s_{x}$ is the standard deviation.

With the intention to eliminate the uninformative descriptors (noise) as well as to prevent model’s over-fitting, a variable reduction on the initial set of descriptors was performed prior to the modelling. For this reason, the descriptors with constant values as well as those with standard deviation less than 0.0001 were removed, as they offer little information for the construction of the model. Additionally, descriptors that are orthogonal to each other were identified by pair-wise correlations using the Pearson correlation coefficient; when two descriptors have an absolute correlation coefficient higher than the desired threshold, only one of them is retained, i.e., avoiding redundancy. Descriptors with absolute pair correlation coefficient value larger than or equal to 0.95 were removed.

To further reduce the chance of correlation among the descriptors, a Kohonen top-map was used [35]. In this way, the rest of the descriptors were mapped onto a network with 7 × 7 architecture of neurons using the transpose of the descriptor matrix; two descriptors were selected from each neuron, the ones with the largest and the shortest Euclidean distance to the central neuron, giving a final set of 96 molecular descriptors for further use.

### 3.3. Selection of Training (TR), Test (TE) and Validation (V) Sets

The methodology used for splitting the dataset is fundamental in order to obtain consistent results; this one must guarantee that the TR set incorporates all sources of expected variability. The most diverse samples should be included into the TR set and be selected in such a way that they are as representative as possible of the global dataset.

The global dataset was divided into TR, TE and V set based on clusters formed in the top-map of the Kohonen neural network. For this purpose, the entire dataset was mapped onto the network using the 1229 2D molecular descriptors calculated. The information space covered by the whole map should be well represented in every subset and to fulfil this requirement, the compounds selected were distributed over the entire Kohonen top-map.

In order to get the best distribution of the objects in the Kohonen top-map, the technical parameters of the network were adjusted, including the network size, the number of learning epochs (training iterations) as well as the maximal and minimal learning rates. Fixed parameters of the network were non-toroidal boundary conditions and triangular function of the neighbourhood. The selection criterion of the best network for splitting purposes was the minimal average error at one object at the maximal neurons’ occupancy.

**Figure 10:**
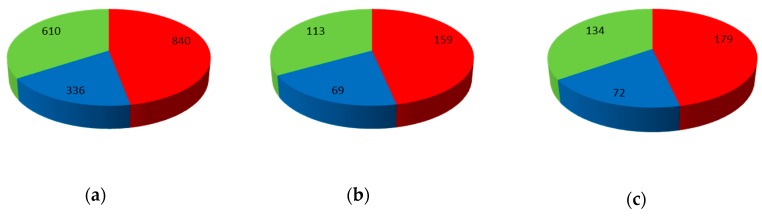
Data set distribution: (**a**) Training set (TR), (**b**) Test set (TE), and (**c**) Validation set (V). Red slice represents P-gp inhibitors; blue slice represents P-gp substrates and green slice represents non-active compounds.

The global dataset was mapped onto the network. The V set containing 385 compounds was selected and not used during model construction and optimization procedures. The TR and TE sets were selected from the rest of the compounds; 1786 compounds were chosen for TR set and 341 for TE set (see Figure 10). The network parameters used for mapping of the dataset were: 20 × 20 neurons, 100 learning epochs, maximal learning rate 0.5 and minimal learning rate 0.01.

Since, the formation of the clusters in the Kohonen top-map is based on unsupervised learning methodology, the resulting distribution in the top-map is influenced only by the structural descriptors used, e.g., percentage of H atoms and the number of secondary amides (aromatic); the clusters are formed as a result of the structural similarity of the objects.

### 3.4. Feature Selection (Variable Selection)

With the intention to construct a classification model grounded on the most significant variables (molecular descriptors), and consequently to optimize its predictive ability, robustness, and reliability, feature selection was performed on the entire final set of 96 descriptors using the genetic algorithm (GA) [28] coupled with counter-propagation artificial neural networks (CP-ANNs) [18].

A population of 95 chromosomes (binary vectors) evolving in 150 generations (iteration steps) was considered in several combinations of different networks and GA parameters. Several GA runs with different random origins were carried out. Fixed parameters were maximal (0.6) and minimal (0.001) learning rate, number of survivals (20) and per cent of mutations (0.02). On the other hand, the number of neurons, number of learning epochs, and number of (initial) genes were modified in order to optimize the selection of variables.

### 3.5. Construction of the Model

**Figure 11:**
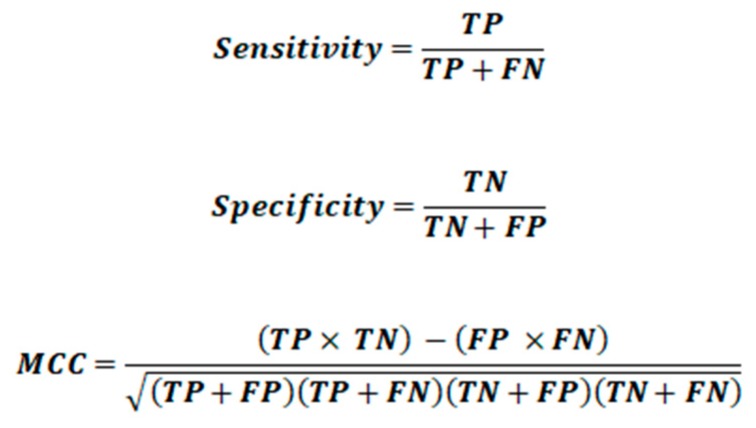
Primary measures related to single classes. TP is the number of true positives, TN is the number of true negatives, FP is the number of false positives and FN is the number of false negatives.

For the construction of the classifier the following steps were carried out:

(1) The dataset was unified in a single sdf file format which contained the structural information and the experimentally determined class (substrate, inhibitor or non-active compound) for each molecule.
(2) 2D molecular descriptors were calculated for the entire dataset using the Dragon 7.0 software [36].
(3) The dataset was divided into TR, TE, and V sets using the Kohonen map clustering method. The V set was excluded and not considered in the training process.
(4) An initial reduction in the number of descriptors was made using a Kohonen top-map.
(5) The TR set was used to construct and train several CP-ANN models; the TE set was used to tune the hyperparameters of the classifier.
(6) Models were optimized using the GA in order to select the most informative variables to include. The model with the best predictive ability was chosen on the basis of the product of the Mathew correlation coefficient (MCC) values (Figure 11) of the TE and TR sets (MCC_TR_*MCC_TE_).

### 3.6. Methods

#### 3.6.1. Self-Organizing Maps (SOM) or Kohonen Artificial Neural Networks

Kohonen artificial neural networks (KANNs) [18] were used for the selection of the TR, TE, and V sets, as well as for the variable reduction (descriptors). KANNs, widely known as self-organizing maps (SOM), are a type of artificial neural network (ANN) that can be used for clustering and visualization tasks. The outcome of the KANN is the mapping of multi-dimensional information into a two-dimensional plane of neurons, based on the similarity among the objects.

The input for KANNs is a vector of independent variables (descriptors) $X_{s}\;=\left(x_{s1},\;x_{s2},\;x_{si},\ldots ,\;x_{sm}\right)$ and the winning neuron $W_{c}$ is the neuron with the weights closest to the input according to the calculated Euclidean distance (4):

(4) $$d_{j}=\sqrt{\overset{m}{\underset{i=1}{\sum }}{\left(x_{si}-\;w_{ji}\right)}^{2}}$$

The next step consists of the correction of the weights of the winning neuron and all the neurons in the range of the topological distance 0> D < D_max_, in order to make them more similar to the input variable, using the following Equations (5)–(7):

(5) $$w_{ji}^{new}\;=w_{ji}^{old}+\Delta w_{ji}$$

(6) $$\Delta w_{ji}=\;\eta \left(t\right)a\left(D_{c}-D_{j}\right)\left(x_{i}-w_{ji}^{old}\right)$$

(7) $$\eta \left(t\right)=\left(a_{max}-a_{min}\right)\frac{t_{max}-t}{t_{max}-1}+a_{min}$$

where the parameter η is the learning rate that has a maximum value at the beginning, i.e., a minimum value at the end of the learning process, $\left(D_{c}-D_{j}\right)$ is the topological distance between the central neuron $W_{c}$ and the current neuron *j* function.

**Figure 12:**
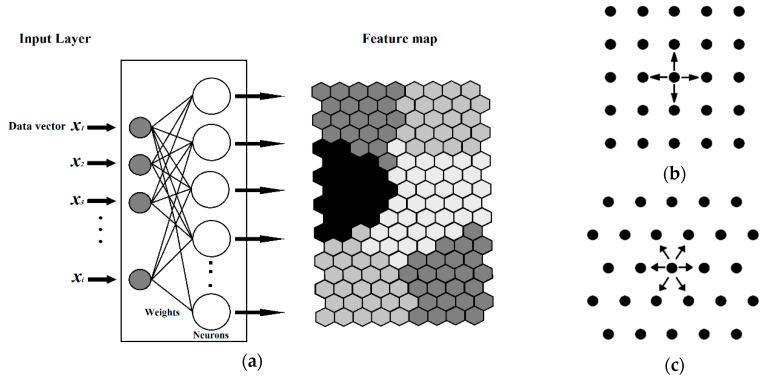
(**a**) Diagram of a Kohonen artificial neural network, (**b**) Square layout of neighbors (S), and, (**c**) Hexagonal layout of neighbors.

The Kohonen type of net is based on a single layer of neurons arranged in a two-dimensional plane that has a well-defined topology. A defined topology means that each neuron has a defined number of neurons as nearest neighbours, second-nearest neighbours, etc. (Figure 12). The neighbourhood of a neuron is usually arranged either in squares or in hexagons, which means that each neuron has either four or six nearest neighbours.

The learning process in the Kohonen neural network is an unsupervised competitive learning and it uses a neighbourhood function to preserve the topological properties of the input space. During the learning process, the positive feedback will extend from the central (winning) neuron to other neurons in some finite neighbourhood around the central neuron. The aim of the Kohonen learning is to map similar signals to similar neuron positions on the network.

#### 3.6.2. Counter-Propagation Artificial Neural Network

**Figure 13:**
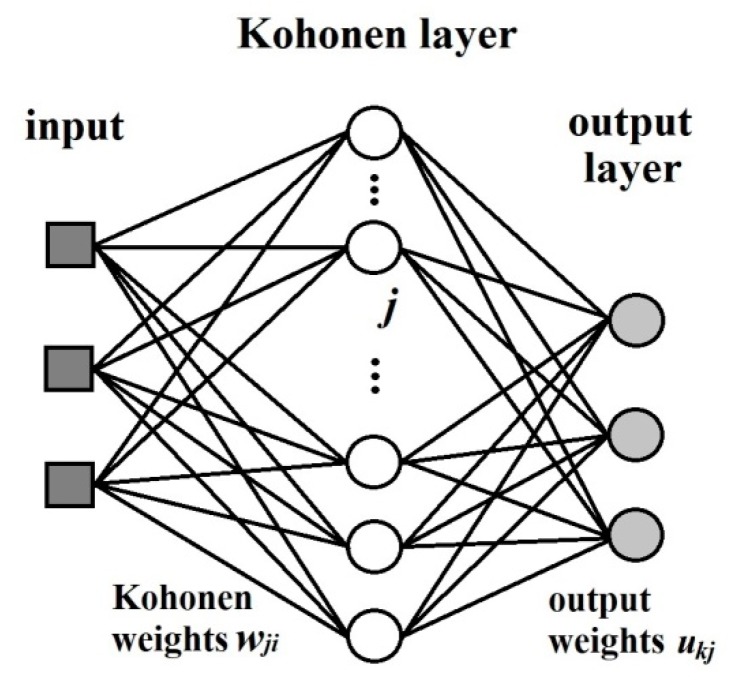
The layout of a counter-propagation artificial neural networks (CP ANNS).

The counter-propagation artificial neural networks (CP ANNs) [18] method was used for building of the model. This learning strategy is based on supervised competitive learning and it requires a set of object-target pairs of data for training and verification. The architecture is basically a two-layer network consisting of a Kohonen layer (influenced by the inputs, i.e., independent variables) and an output layer (influenced by the targets, i.e., dependent variables) (Figure 13). The inputs are fully connected to the Kohonen network, where competitive learning is performed, i.e., each unit in the input layer is linked to all neurons in the Kohonen Layer. In Figure 13 the weights connecting the input unit *i* with the Kohonen neuron *j* is label as $w_{ji}$, each neuron in the Kohonen layer is described by a weight vector $W_{j}$. The neurons of the Kohonen layer are connected to the neurons in the output layer. This is a full connection. However, after each input, only a certain neighbourhood of a given neuron is connected to the output neurons, and only the weights linking these neurons are allowed to change. The weights connecting the *j*-th neuron in the Kohonen layer with th *k*-th neuron in the output layer are labelled $u_{kj}$, and the weight vector belonging to a given output neuron is labelled $R_{k}$. A given answer is not stored as a set of weights in one neuron, but as one component of the weights of all the output neurons. This kind of organization requires the number of neurons in the Kohonen layer to be equal to the number of answers to be stored, and the number of neurons in the output layer to be equal to the number of variables comprising the output answer, e.g., one thousand answers, each consisting of four variables, requires a Kohonen network with one thousand neurons, and an output layer with four. The input layer should have the same number of units as input variables.

The training process is very similar to the KANNs. First the objects are mapped in the Kohonen layer in an unsupervised manner and then the supervised learning is used for the correction of weights in the output layer. The weights are adapted by comparing the actual output with an ideal output. The weights in the output layer are influenced by the position of the winning neuron in the input layer, as it defines the neighbourhood in the output layer, and by the target values. Once the winning neuron has been selected, two types of correction are made: first, the correction of weights $w_{ji}$ within the neurons of the Kohonen layer, and secondly, the correction of weights in the output layer $u_{kj}$ according to the following Equation (8):

(8) $$\Delta u_{kj}=\;\eta \left(t\right)a\left(D_{c}-D_{j}\right)\left(y_{i}-u_{kj}^{old}\right)$$

The outcome of the learning process is an arrangement of objects in a two-dimension map that corresponds exactly to the maps generated in the Kohonen layer. The output is taken from all weights between one Kohonen neuron and all the output neurons and there is a one-to-one correspondence between the neurons in the Kohonen map and those in the output map. CP ANNs can be used for building models able to predict unknown properties of new objects. It is also a suitable tool for clustering and classification tasks.

#### 3.6.3. Genetic Algorithm

The genetic algorithm (GA) [28] was used in combination with CP ANNs for the descriptors selection in order to improve the model performance. This algorithm of heuristic search reflects the process of natural selection, where the fittest individuals are selected for reproduction in order to produce offspring of the next generation.

Implementing to a multi-dimensional descriptor space, GA can effectively be used for descriptors selection and/or the optimal adjustment of parameters, which must be passed to a function that evaluates how well they solve the problem. The general steps for descriptor selection utilizing GA are as follows:

(1) Create an initial population of genetic vectors and calculate their fitness.
(2) Choose two members of this population based on their fitness to become parents.
(3) Use a mating operator to construct a new genetic vector from the parents.
(4) Use a mutation operator to probabilistically change the genetic vector.
(5) Calculate the fitness of this offspring and have it replace the weakest member in the population.
(6) Return to Step 2 until a sufficient number of offspring has been produced.

#### 3.6.4. Applicability Domain

The applicability domain (AD) of (Q)SAR models is defined by the Organization for Economic Co-Operation and Development (OECD) as the response and chemical structure space in which the model makes predictions with a given reliability. It is defined as a “physicochemical, structural, or biological space, knowledge or information on which the training set of the model has been developed, and for which it is applicable to make predictions for new compounds” [37]. The purpose of defining the AD for a given (Q)SAR model is to determine the prediction accuracy of a new unknown compound independently of our naïve interpretation of its similarity to the molecules from the set used to construct and validate the model.

There are different approaches for describing the AD of a model: methods based on ranges of molecular descriptors, geometrical methods, distance-based methods, and probability distribution-based methods. Within distance-based approaches, three are widely used in (Q)SAR research: Euclidean, Mahalanobis and city-block distance. The AD of the model in this study was analysed using the ED between objects (molecules) and the central neuron of the neural network.

The ED between the molecules and the central neuron (in the Kohonen layer of CP ANN models) is the fundamental characteristic of the neural network. It represents the interval between a central node (*ci*) in the Kohonen layer and an input pattern (X). The ED can be expressed using the following Equation (9):

(9) $$ED(X,w_{ci})=sqrt\;\left((x_{1}^{T\;}-w_{ci1}{)}^{2\;}+(x_{2}^{T\;}-w_{ci2}{)}^{2\;}+\cdots +(x_{m}^{T\;}-w_{cim}{)}^{2\;}\right)$$

where $w_{ci1}$, $w_{ci2}$,…, $w_{cim}$ are the weights to the neuron ***ci*** corresponding to a particular descriptor, and ***m*** is the number of descriptors or levels corresponding to a particular descriptor in the Kohonen layer. Input patterns for each level can be expressed as transposed matrixes. Each transpose matrix ($x_{1}^{T\;},\;x_{2\;}^{T\;},\;\ldots ,\;x_{m}^{T})$ includes the values of descriptors $D_{1},\;D_{2},\ldots ,D_{m}$ respectively, calculated for each molecule. The distances are unitless because the descriptors have been previously autoscaled.

The goal of an AD is to set up boundaries whereby the obtained predicted values can be trusted with confidence. However, there is not a clear consensus about the determination of thresholds in AD for non-linear classification models [38]. Since, our model is a non-linear one we did not fix a warning threshold, but look for the prediction accuracy of the models in the chemical and descriptors space, and tried to find out the space where models gave reliable predictions.

A very important issue that should be taken into account during the determination of the AD is the uncertainty. There may be input uncertainties, and variability and structural (model) uncertainties, which derive from simplifications of the reality due to limited systematic knowledge. As a result of uncertainties associated with individual (Q)SAR predictions, some predictions may fall within the defined AD of the model, but be unreliable due to properties and features not accounted for by the model. On the other hand, a chemical falling outside the defined AD, may still exhibit the response being modelled, because it brings out this response by a mechanism not accounted by the model under study. The essential problem of AD definition is to find out the uncertainty areas where less reliable predictions fall.

## 4. Conclusions

In summary, a multi-class classification model for P-glycoprotein inhibitors, substrates and non-active compounds was developed, which provided a good classification performance evidenced by the values of the global indices’ non error rate (NER) and the average precision (AvPr) for the training (TR), test (TE), and validation (V) sets. Hence, the presented classifier could be used as a reliable in silico screening tool for identifying potential ligands of P-glycoprotein (P-gp). Unlike classifiers presently available, the one developed here not only separates active/non-active compounds, but differentiates substrates and inhibitors. This is an important detail that would extend the use of the multi-class classifier to different purposes. The fast screening tool is supposed to be used as an initial step in assessing a set of molecules of interest, even before implementing a molecular modelling method (e.g., molecular docking and molecular dynamics simulations, which are more demanding and time consuming). After the initial fast screening, the molecules that show a desirable response would be subjected to further detailed studies, including final experimental testing of their interactions with the P-gp.

## Figures and Tables

**Table 1:** 26 Dragon descriptors selected for the model.

Symbol	Definition	Block Description
H%	percentage of H atoms	Constitutional indices
nR07	number of 7-membered rings	Ring descriptors
D/Dtr11	distance/detour ring index of order 11	Ring descriptors
MWC01	molecular walk count of order 1	Walk and path counts
X2A	average connectivity index of order 2	Connectivity indices
SIC3	Structural Information Content index (neighborhood symmetry of 3-order)	Information indices
VE1sign_B(s)	coefficient sum of the last eigenvector from Burden matrix weighted by I-state	2D matrix-based descriptors
ATSC7m	Centred Broto-Moreau autocorrelation of lag 7 weighted by mass	2D autocorrelations
MATS6v	Moran autocorrelation of lag 6 weighted by van der Waals volume	2D autocorrelations
GATS4s	Geary autocorrelation of lag 4 weighted by I-state	2D autocorrelations
P_VSA_LogP_3	P_VSA-like on LogP, bin 3	P_VSA-like descriptors
P_VSA_ppp_D	P_VSA-like potential pharmacophore points, D-hydrogen-bond donor	P_VSA-like descriptors
nRCOOR	number of esters (aliphatic)	Functional group counts
nArCONHR	number of secondary amides (aromatic)	Functional group counts
nArCO	number of ketones (aromatic)	Functional group counts
H-048	H attached to C2(sp3)/C1(sp2)/C0(sp)	Atom-centred fragments
SdsCH	Sum of dsCH E-states	Atom-type E-state indices
CATS2D_01_DN	CATS2D Donor-Negative at lag 01	CATS 2D
CATS2D_05_PP	CATS2D Positive-Positive at lag 05	CATS 2D
CATS2D_02_PL	CATS2D Positive-Lipophilic at lag 02	CATS 2D
B07[O-F]	Presence/absence of O - F at topological distance 7	2D Atom Pairs
F01[C-C]	Frequency of C - C at topological distance 1	2D Atom Pairs
F02[C-O]	Frequency of C - O at topological distance 2	2D Atom Pairs
F04[C-P]	Frequency of C - P at topological distance 4	2D Atom Pairs
F04[C-Br]	Frequency of C - Br at topological distance 4	2D Atom Pairs
F07[O-F]	Frequency of O - F at topological distance 7	2D Atom Pairs

**Table 2:** Statistical performance of the selected model.

Global Indices	Training Set			Test Set			Validation Set		
NER	0.93			0.85			0.70		
AvPr	0.93			0.87			0.70		
	**Training set**			**Test set**			**Validation set**		
	**I ^1^**	**S ^2^**	**NA ^3^**	**I**	**S**	**NA**	**I**	**S**	**NA**
Sensitivity	95.1	92.5	91.6	90.5	79.7	85.8	75.9	65.2	69.4
Specificity	95.6	97.8	96.7	91.2	97.4	92.1	83.0	90.7	83.2
MCC	0.90	0.89	0.88	0.79	0.78	0.76	0.63	0.54	0.52

^1^ Inhibitors. ^2^ Substrates. ^3^ Non-actives.

**Table 3:** Confusion matrix for the Validation set of 385 compounds.

		**Experimental Class**	
		**Inhibitors**	**Non-Inhibitors**
**Predicted class**	**Inhibitors**	136	35
	**Non-Inhibitors**	43	171
		**Substrates**	**Non-Substrates**
	**Substrates**	47	29
	**Non-Substrates**	25	284
		**Non-Active**	**Active**
	**Non-Active**	93	42
	**Active**	41	209

**Table 4:** CP-ANN parameters for the selected model.

Parameters of CP-ANN			
Network Dimension	Learning Epochs	Max. Learning Rate	Min. Learning Rate
43 × 43	600	0.6	0.001

## Supplementary Materials

The following are available online, Excel file S1: P-gp data set, Excel file: Descriptors values for each set, Excel file: Weights for the final CPNN model, Figure S1: Structures of the compounds with largest ED to the central neuron in the TE set, Figure S2: Structures of the compounds with largest ED to the central neuron in the V set, Figure S3. Distribution of the 24 overlapping negative compounds (P-gp non-inhibitor (NI) and P-gp non-substrate (NS)) in the response map of the non-active class.
