# Supplementary material for: Multiclass Classifier for P-Glycoprotein Substrates, Inhibitors, and Non-Active Compounds
Source: Molecules. 2019 May 25;24(10):2006. doi: 10.3390/molecules24102006 (PMC6571636; doi:10.3390/molecules24102006)
Supplement: Supplementary file 1 [file molecules-24-02006-s001.zip › molecules-503177-2nd-revised-supple/Supplementary Materials.pdf]

Supplementary Materials for:

Multiclass Classifier for P-glycoprotein Substrates, Inhibitors, and Non-Active Compounds

Liadys Mora Lagares [1,2], Nikola Minovski [1] and Marjana Novič [1,*]

[1] Theory Department, Laboratory for Cheminformatics, National Institute of Chemistry, Ljubljana, Slovenia; marjana.novic@ki.si, nikola.minovski@ki.si, liadys.moralagares@ki.si
[2] Jozef Stefan International Postgraduate School, Ljubljana, Slovenia
[*] Correspondence: marjana.novic@ki.si; Tel.: +386-01-476-0253

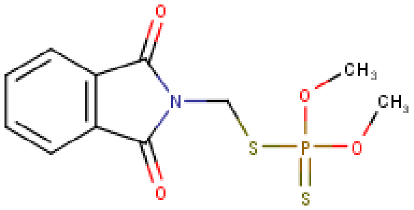

(**a**)

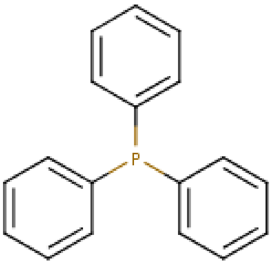

(**b**)

Figure S1. Structures of the compound with largest ED to the central neuron in the TE set: (**a**) Phosmed; (**b**) Triphenylphosphane.

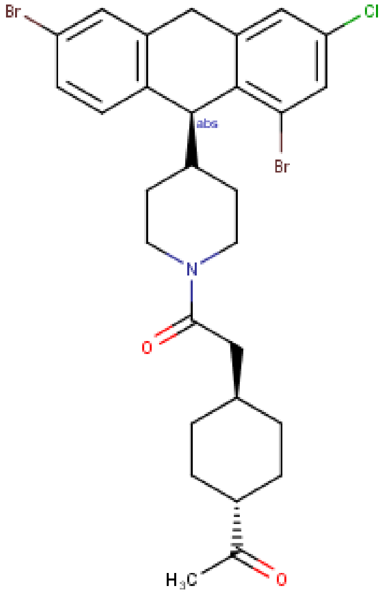

(**a**)

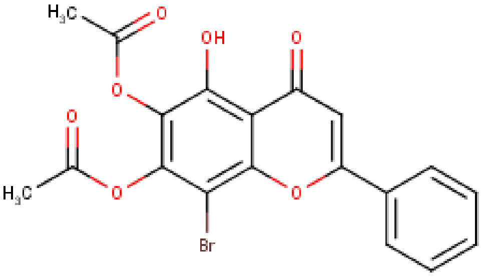

(**b**)

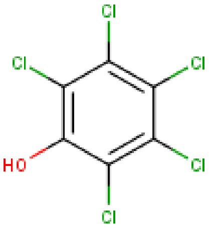

(**c**)

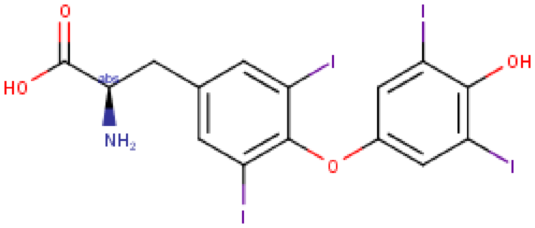

(**d**)

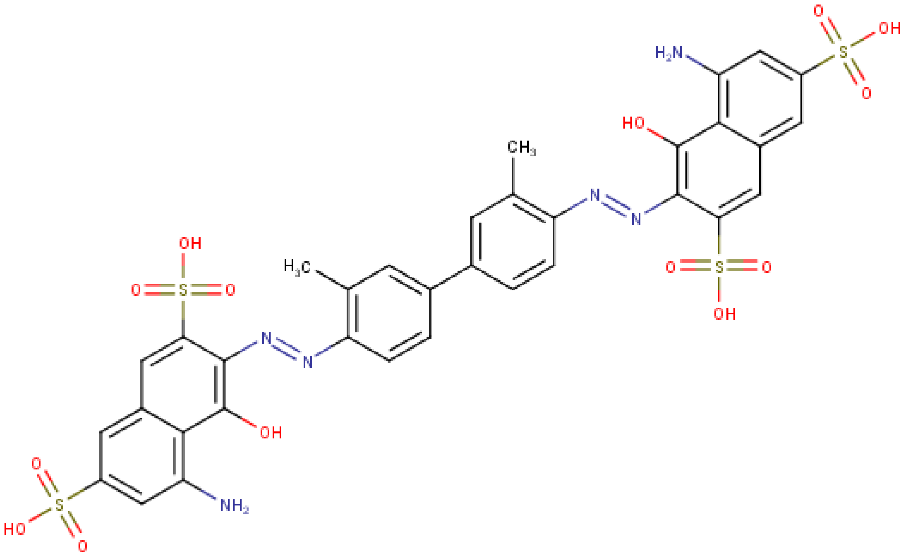

OH
O
S
O
H₃C
N
N
OH
NH₂
O
S
HO
O
CH₃
N
N
H₂N
HO
O
S
OH
O
O
S
O
OH

(**e**)

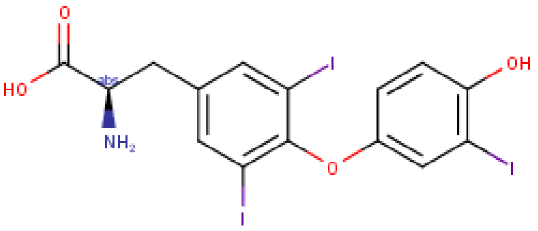

O
HO
NH₂
I
O
I
OH
I

(**f**)

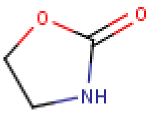

O
O
NH

(**g**)

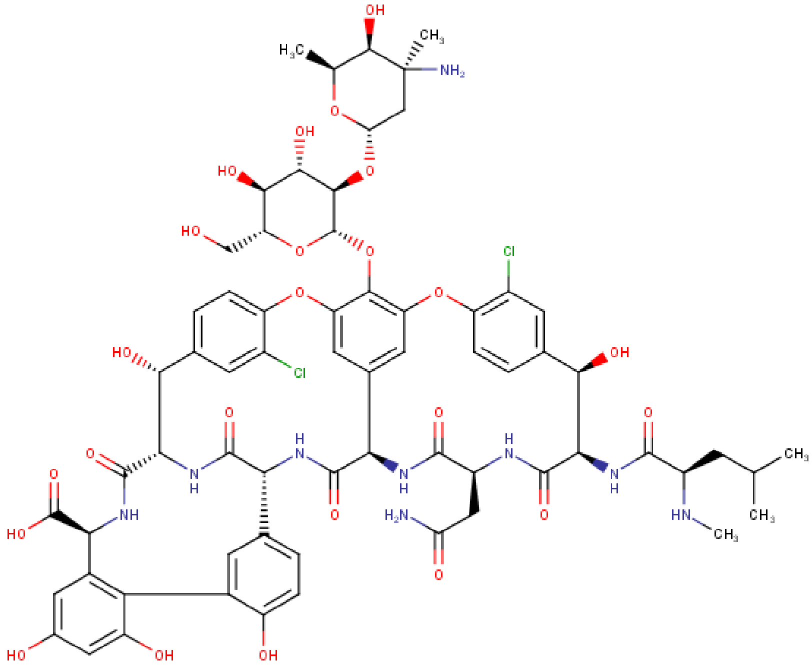

OH
H₃C
CH₃
NH₂
O
OH
HO
O
HO
O
O
Cl
O
O
Cl
HO
OH
O
H
N
O
N
H
O
H
N
O
N
H
O
O
N
H
O
O
HO
NH
N
H
H₂N
O
N
H
HN
CH₃
CH₃
CH₃
HO
OH
OH

(**h**)

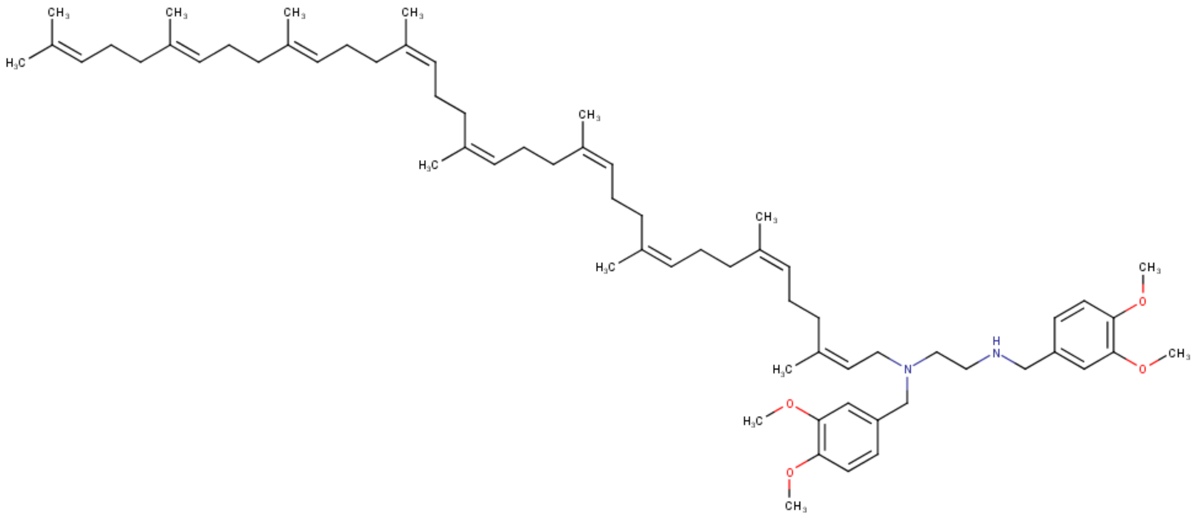

(**i**)

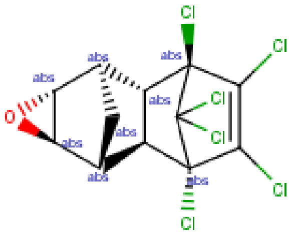

(**j**)

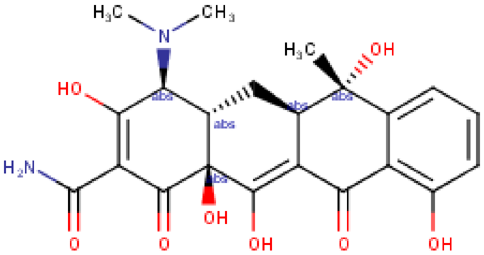

(**k**)

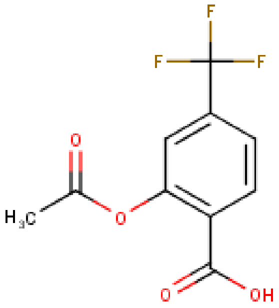

(**l**)

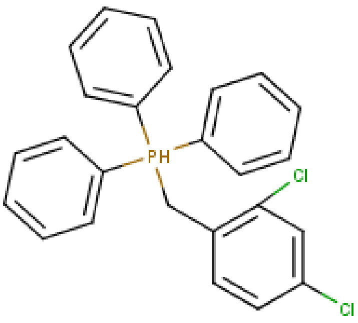

(**m**)

Figure S1. Structures of the compound with largest ED to the central neuron in the V set: (**a**) 2-(4-acetylcyclohexyl)-1-[4-[(9S)-1,6-dibromo-3-chloro-9,10-dihydroanthracen-9-yl]piperidin-1-yl]ethanone; (**b**) (7-acetyloxy-8-bromo-5-hydroxy-4-oxo-2-phenylchromen-6-yl) acetate; (**c**) Pentachlorophenol; (**d**) Thyroxine; (**e**) Trypan blue; (**f**) Triiodothyronine; (**g**) 2-Oxazolidinone; (**h**) Vancomycin; (**i**) Sdb-ethylenediamine; (**j**) Dieldrin; (**k**) Tetracycline; (**l**) Triflusal; (**m**) (2,4-dichlorophenyl)methyl-triphenylphosphanium.

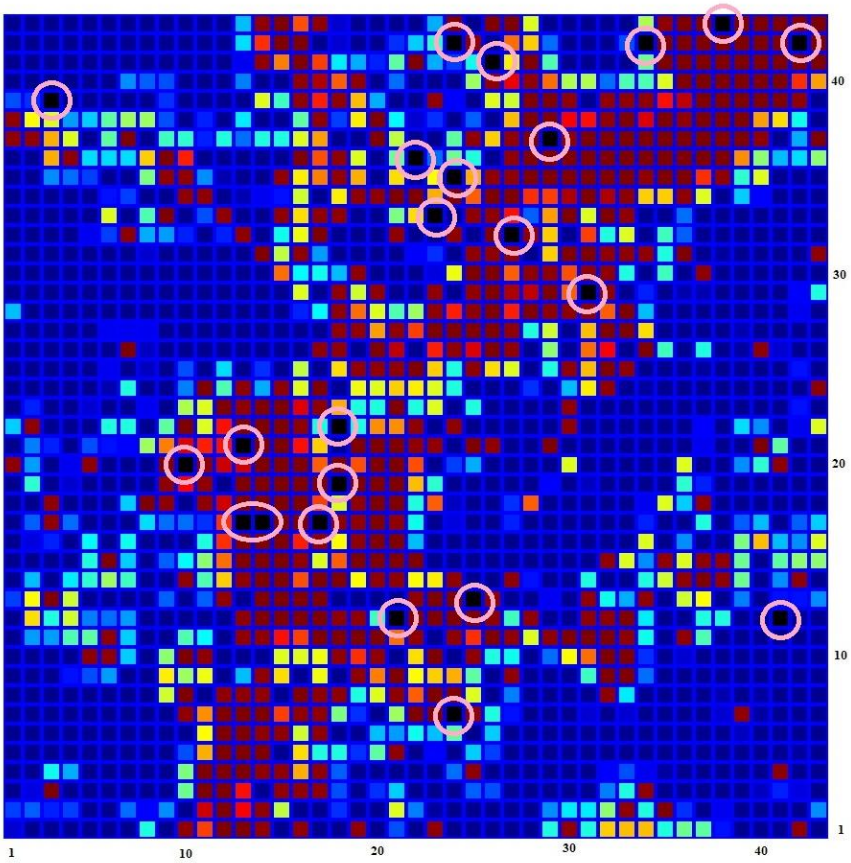

Figure S3. Distribution of the 24 overlapping negative compounds (P-gp non-inhibitor (NI) and P-gp non-substrate (NS)) in the response map of the non-active class. These are the only absolutely certain non-active compounds. The position of the neurons where these compounds are located were coloured black and highlighted with a pink circle. They are well spread over the whole area occupied by the all non-active compounds meaning that structural similarity (which is the only influencing factor for forming clusters in the Counter-Propagation Artificial Neural Network output layer) with other non-actives, tested with only one assay, is significant. The only area of non-active compounds that does not include any of the double-tested non-actives, is around the neuron at the position (13, 5) in lower left part of the map, so we may consider the predictions associated with this area less reliable.
